# An Opportunity for Diagonal Development in Global Surgery: Cleft Lip and Palate Care in Resource-Limited Settings

**DOI:** 10.1155/2012/892437

**Published:** 2012-12-20

**Authors:** Pratik B. Patel, Marguerite Hoyler, Rebecca Maine, Christopher D. Hughes, Lars Hagander, John G. Meara

**Affiliations:** ^1^Program in Global Surgery and Social Change, Department of Global Health and Social Medicine, Harvard Medical School, Boston, MA 02115, USA; ^2^Department of Plastic and Oral Surgery, Boston Children's Hospital, 300 Longwood Avenue, Enders 1, Boston, MA 02115, USA; ^3^Department of Surgery, University of California San Francisco Medical Center, San Francisco, CA 94131, USA; ^4^Department of Surgery, University of Connecticut Health Center, Farmington, CT 06030, USA; ^5^Department of Pediatric Surgery and International Pediatrics, Faculty of Medicine, Lund University, Lund SE-221 00, Sweden

## Abstract

Global cleft surgery missions have provided much-needed care to millions of poor patients worldwide. Still, surgical capacity in low- and middle-income countries is generally inadequate. Through surgical missions, global cleft care has largely ascribed to a vertical model of healthcare delivery, which is disease specific, and tends to deliver services parallel to, but not necessarily within, the local healthcare system. The vertical model has been used to address infectious diseases as well as humanitarian emergencies. By contrast, a horizontal model for healthcare delivery tends to focus on long-term investments in public health infrastructure and human capital and has less often been implemented by humanitarian groups for a variety of reasons. As surgical care is an integral component of basic healthcare, the plastic surgery community must challenge itself to address the burden of specific disease entities, such as cleft lip and palate, in a way that sustainably expands and enriches global surgical care as a whole. In this paper, we describe a diagonal care delivery model, whereby cleft missions can enrich surgical capacity through integration into sustainable, local care delivery systems. Furthermore, we examine the applications of diagonal development to cleft care specifically and global surgical care more broadly.

## 1. Introduction

The inadequacy of surgical and anesthetic capacity in resource-limited settings is well demonstrated [1–5], as is the particular need for more robust pediatric surgical services [6–8]. Total surgical disease burden, estimated at 11–15% of disability-adjusted life years (DALYs) lost worldwide, disproportionately affects low- and middle-income countries (LMICs) [9–11]. Cleft lip and palate (CLP) and other congenital anomalies account for approximately 9% of this burden [9], and the consequences of untreated CLP range from social ostracism to death [12–14]. Although the economic burden of untreated CLP and the value and cost effectiveness of global cleft treatments have been proven [15–17], CLP treatment capacity remains insufficient in LMICs [18, 19]. Historically plastic surgeons' efforts to address this need have focused on short-term, service-oriented commitments—a vertical approach to healthcare [20].

Narrowly focused, disease-specific, and vertical programs tend to operate outside the existing national and local healthcare structures, supplying their own facilities and delivery mechanisms [21–23]. By contrast, the horizontal approach focuses on developing and strengthening existing public infrastructure, with an emphasis on primary care and broadly applicable health interventions [22, 23].

Although humanitarian cleft care missions have provided crucial treatments for many patients in LMICs who would not otherwise have had access to care, there is an untapped potential in optimally channeling the resources and skills of mission groups into sustainable, local care-delivery systems. As the plastic surgery community continues to evaluate the ideal role of missions in providing comprehensive cleft care in LMICs, an emphasis should be placed on the concept of diagonal development: an integration of vertical and horizontal approaches in a way that enriches the overall educational and surgical capacity of LMICs. In this paper, we explore the benefits and limitations of the vertical and horizontal approaches to healthcare delivery (Table 1) and apply that framework to global CLP care.

## 2. Horizontal and Vertical Approaches: Benefits and Limitations

### 2.1. Vertical Programs

The vertical approach to global health is disease specific and has been a particularly common approach among global infectious disease initiatives [22, 24, 25]. Proponents cite milestones like the eradication of smallpox and dramatic decreases in rates of new HIV infection as evidence of vertical intervention success [22, 26]. Vertical interventions are relatively scalable and are thus ideal for urgent humanitarian responses to disasters or epidemics, which have traditionally garnered significant attention from donors [27]. Additionally, vertical programs efficiently deliver necessary surgical supplies and equipment for disease-specific use in LMICs [28].

However, the vertical approach may also yield parallel and uncoordinated interventions, detract attention from the systemic weakness of national healthcare institutions, compromise countries' autonomy and participation in healthcare initiatives, alienate patients whose healthcare needs exceed the narrow range of provided services, and divert funds from other important causes of morbidity and mortality [22, 29, 30]. The vertical approach has also been criticized for not adequately developing the infrastructure and workforce necessary to address even disease-specific needs, letting alone broader healthcare demands [31–33]. Finally, given the complexity of socioeconomic and environmental disease determinants, narrow vertical efforts, which are not designed to address these issues, may be less effective or even harmful to the populations they aim to serve [22, 24].

### 2.2. Horizontal Programs

The horizontal approach to healthcare delivery emphasizes long-term investments in healthcare infrastructure and the expansion of publicly funded healthcare systems [20, 34, 35]. Examples include WHO efforts to strengthen primary care systems and World Bank-guided reforms of district-level health administrations [21, 27, 36]. Although the horizontal model of public health interventions preceded the vertical model [20] it has seen renewed emphasis in recent years, particularly as infectious disease treatment groups such as PEPFAR, Human Resources for Health (HRH), and The Global Fund to Fight AIDS, Tuberculosis, and Malaria, transition away from strictly vertical models [21]. Proponents of the horizontal model even argue that disease-specific therapies can be delivered most efficiently through a functional primary healthcare system. Furthermore, horizontal approaches have greater potential to address patients' comorbidities and other health needs, and they intentionally strengthen healthcare systems for the benefit of all current and future patients [21]. As surgery is increasingly acknowledged as an integral part of healthcare worldwide [3], the horizontal model has begun to be applied to surgical disease, through investments in surgical infrastructure and human capital [37].

In surgery and in other domains, however, horizontal development has been hindered by concerns regarding the scope and time frame of horizontal interventions. Horizontal initiatives take extended periods of time to be implemented, may be less suitable to humanitarian emergencies, and depend heavily on governmental legitimacy and functionality in order to be effective [27]. Additionally, defining objective metrics for success in horizontal interventions may be particularly challenging due to larger patient cohorts and diverse causes of morbidity and mortality [21]. For all of these reasons, horizontal development has been of limited appeal to private funding organizations [21]. Lastly, horizontal projects may seem incompatible with the domestic commitments of many global health-oriented physicians in practice in wealthy countries.

### 2.3. Diagonal Programs

**Figure 1:**
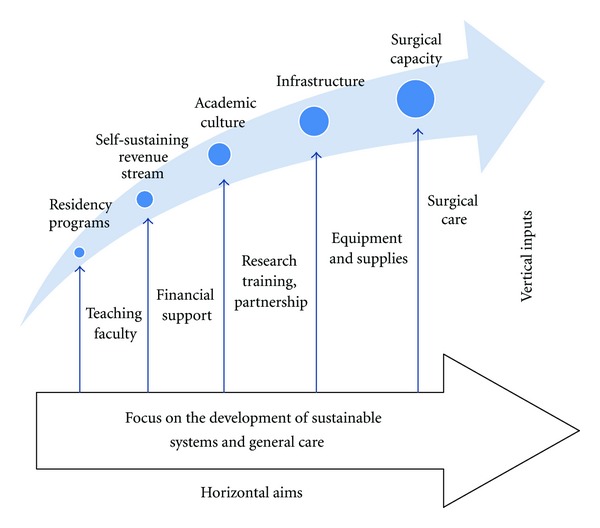
A diagonal approach harnesses the power of horizontal aims and vertical inputs.

“Diagonal” approaches refer to programs which are neither purely vertical nor purely horizontal [34, 35]. Rather, these programs find synergy between the immediate advantages of vertical inputs and the long-term benefits of horizontal aims, ultimately increasing access and enriching capacity of surgical services (Figure 1).

Diagonal interventions are becoming increasingly common [21], particularly as the horizontal approach is recognized as an effective means of delivering disease-specific care [24, 27]. In addition to infectious disease [38], family planning and maternal and child health are areas in which the integration of vertical and horizontal care has been reported [11, 39, 40]. Although the vertical approach may also yield positive “spill-over,” in which focused health initiatives in one disease area or population also benefit the health system as a whole [41]; the diagonal approach embraces these broader impacts as a primary aim instead of as a welcome externality.

## 3. Building Capacity While Addressing Specific Needs: A Diagonal Approach to Global CLP Care

The traditional “missions” model of cleft care rests partially on the premise that one-time interventions can effectively treat craniofacial anomalies, that they produce a high return for time and resources invested, and that they are feasible commitments for visiting providers [18, 42–44]. However, as cleft palate missions have grown in scale and scope, they have demonstrated a willingness to think critically about their care delivery models. As a result, many cleft treatment groups have begun to integrate vertical and horizontal approaches in order to maximize their positive impact and minimize any negative consequences [42, 43, 45–49]. In the case of Interplast, these changes include an emphasis on local partnerships with the explicit goal of creating self-sufficient, independently functioning local sites [50]. Operation Smile and others have evolved from purely vertical care providers to integrated system creators through a continued emphasis on mindful and reflective practice [18].

We build upon these shifts in global cleft care delivery and argue that a diagonal approach can build on the strengths of the vertical model, while also addressing its weaknesses. In the case of global CLP repair, diagonal programs would retain the focused services and resource inputs of surgical missions, while incorporating efforts to expand surgical capacity, increase human capital, and provide comprehensive care in general. As a result, through diagonal development, surgical care in LMIC can be broadly enriched in several key deliverable areas (Table 2).

### 3.1. Integrated, Longitudinal CLP Care

The optimal care of patients with cleft lip and palate patients is complex, longitudinal, and interdisciplinary [51–54]. Children with cleft anomalies benefit from dental and orthodontic services, speech therapy, otologic care, and occasionally revision surgeries [9, 55, 56]. In many wealthy nations, participation in integrated cleft centers allows for parental education and support often from the time of prenatal diagnosis through the postoperative care. Cleft centers provide not only the essential followup to identify and address surgical complications, but also permits tracking of long-term outcomes to support general quality improvement projects. Multidisciplinary services are frequently lacking in LMICs, and often access to long-term followup is limited as well. Unfortunately these same patients in LMICs face increased risk for surgical complications due to higher incidences of malnutrition and concurrent illness [23, 44].

The traditional vertical structure of CLP missions is not well suited to longitudinal, integrated CLP care in LMICs [57], especially because effective delivery of these services may require multiple visits that are beyond the scope of a purely vertical treatment model [14, 58]. Adopting a diagonal approach to cleft palate care would address many of these limitations by transitioning care from fragmented efforts of visiting providers to a more sustained local physician practice over the long term. This could be achieved either by a limited permanent staff complemented by frequent missions or by a constant rotation of visiting teams with no coverage gaps. Additionally, specialized cleft centers, similar to those described in wealthy nations, have been described as feasible and sustainable delivery models to ensure comprehensive CLP care in LMICs [59]. The diagonal approach takes this concept one step further, by integrating specialized surgical care into the longitudinal services of local healthcare systems and structures. This can foster trust in and utilization of the local healthcare system, both by CLP patients and their families and community members.

The logistical challenges of contacting and locating former patients, varying degrees of patient compliance, especially in the setting of insufficient patient education, and coordinating followup with local professionals [14, 56, 60–63] have been barriers to follow up on vertical missions. Follow-up rates have been correspondingly low: among medical mission groups that do provide postoperative care, rates range from 5% to 35% of patients [58]. Cleft missions groups have made progress in monitoring surgical outcomes, for instance through the development of outcomes databases [18, 43, 49, 62]. However, an increased focus on outcomes is needed. One paper examining the long-term results of palatoplasties, performed by local and visiting surgeons in Ecuador, found a significantly higher rate of fistula formation among Ecuadorian patients than among their counterparts in wealthy countries [56]. Other international researchers have noted that palatal dehiscence and residual or recurrent fistulae were frequently encountered during their studies, even when palate integrity was not an outcome in question [60]. Through its emphasis on longitudinal care, diagonal development provides an optimal framework for outcomes research, which is essential to identify and address the underlying causes of these troubling results. Furthermore, diagonal approaches can facilitate outcomes monitoring through emphasis on general, not disease-specific, infrastructure improvements, such as electronic medical records, clinical measurement tools, and a culture of medical documentation and outcomes-driven practice. Once in place, these infrastructure improvements could help optimize quality and safety of all clinical care delivery, including CLP treatments.

Improved infrastructure for followup and outcomes monitoring through a diagonal delivery model can also improve access to the variety of specialists needed to provide comprehensive CLP care. While some programs have implemented interdisciplinary, comprehensive services for cleft patients in LMICs [64], for instance by using telemedicine to provide speech therapy [47, 65, 66], this type of care depends on broad manpower and healthcare structure capabilities, neither of which is the focus of a vertical model. By reinforcing the importance of surgical care, a particular strength of the vertical approach, while simultaneously building capacity for other necessary CLP services, the diagonal model has the potential to improve the outcomes of patients in LMICs. Additionally, effective patient education regarding the comprehensive treatment options for CLP would result from utilization of the significant outreach capacity of the existing healthcare system.

An investment and focus on interdisciplinary care would also benefit resource-limited communities at large. Many children and adults without cleft anomalies have need for services like speech therapy and audiologic care, in addition to surgery and anesthesia. If promoted as a component of CLP care in LMICs, these services could be available for patients with and without CLP. In addition, each service would represent a channel by which patients could seek their first contact within the broader healthcare system, promoting a culture of individual health agency.

By providing long-term follow-up of cleft patients, supporting the growth of essential complementary services and developing quality improvement projects through outcomes monitoring, the diagonal model of cleft care delivery would help achieve the ethical goal of providing the same treatment to patients in poor countries which is the standard of care in wealthy countries.

### 3.2. Equitable Access

Equitable access results from a focus on patient-centered interventions that are able to prioritize diverse patient needs without the logistical constraints of short-term missions. In wealthy countries, standard of care for patients with cleft anomalies involves careful timing of surgical correction. Patients treated on annual missions have a lower likelihood of being operated on within recommended windows [63], which can impact surgical outcomes, development of facial structures, speech, and hearing [56, 67, 68]. Longitudinally focused, diagonal efforts remove the need to operate based on visiting provider availability, allowing for interventions at the appropriate developmental stage, and enabling more timely and equitable cleft care.

Inadequate physical access—lack of transportation or long travel distances to care facilities—is a significant barrier to care for patients in resource-limited settings [69]. Improved patient transportation and high-quality facilities can help reduce morbidity and mortality attributable to multiple causes in LMICs [70]. A diagonal model could address these barriers, for instance by devoting funds and resources to patient transportation and lodging needs. One might also envision a health-services bus, perhaps integrated with existing local public transport infrastructure, which would travel to remote communities in order to provide regularly-scheduled medical care to patients there. For cleft patients, this service would facilitate initial assessments, transportation to the surgical center, and followup, as needed. For all patients, this service would provide a critical link between communities, clinics, and hospitals, promoting equitable access to care.

### 3.3. Local Workforce Development

Cleft missions have been criticized for undermining the efforts and authority of local providers [71]. Importing surgical services may suggest to patients and community members that surgery requires large teams and equipment that may not be accessible locally. This diminishes confidence in local providers, lowers professional morale, and decreases revenue for local facilities as patients with means to pay for local providers to perform their operations rely on visiting surgeons [72].

In response to these criticisms, many cleft missions groups have adopted the training, promotion, and support of local surgeons as a primary objective [58, 73]. However, this method often consists of choppy, “on the spot” intraoperative lectures by the visiting surgeon, with little structure regarding teaching objectives, and even less time for reciprocal teaching by host providers [58]. This unilateral approach to knowledge transfer undermines the important contributions of local surgeons and other healthcare professionals, who are critical to all steps of care delivery and capacity building in resource-limited settings and whose clinical skills are well adapted to the local resource limitations and epidemiology of disease [71]. Additionally, a lack of structure regarding teaching objectives likely impairs the systematic mastering of skills and knowledge for both local and visiting surgeons [74]. Finally, an educational focus on surgeons as providers of cleft care strictly misses the opportunity to train other essential perioperative and interdisciplinary staff—pediatric anesthesiologists, surgical intensivists, scrub technicians, operating room nurses, and speech therapists—in the provision of safe and comprehensive cleft care.

More robust academic partnerships, as fostered by diagonal development, would also promote local academic leaders and would enhance training programs for numerous types of healthcare providers. In particular, greater numbers of well-trained surgeons, scrub technicians, nurses, and anesthesiologists would improve surgical care for all patients in LMIC; cleft patients are included.

### 3.4. Equitable Trainee Experiences

It is not uncommon for general or plastic surgery residents from wealthy countries to complete an international rotation in LMICs [75, 76]. Research shows that these experiences enhance the training of the visiting surgical residents [77, 78], but it is less clear that host institutions benefit equally. As institutional partnerships between academic centers in wealthy LMIC become increasingly common [10, 37], the cleft care community, and the surgical community at large, must look beyond the needs of visiting trainees to the needs of students, residents, faculty and staff in host institutions and communities [79].

Diagonal development in cleft care can facilitate equity between visiting and local trainees and providers through its longitudinal view of the surgical care. For instance, the educational objectives for rotating trainees from wealthy countries could perhaps center on tackling systems-based and logistical challenges of surgical care delivery in resource-limited environments, in addition to the acquisition of operative experience. Visiting trainees could be mentored and instructed in these areas by local providers with expertise in local systems and care-delivery challenges. Furthermore, visiting trainees could complement the experiences of local trainees by enabling local residents to spend more time with visiting faculty, learning clinical skills through intraoperative teaching sessions, formal lectures, and skills-based workshops. This approach would benefit local trainees and, ultimately, the patients they will serve in their home communities.

### 3.5. An Academic Culture of Investigation and Empowerment

A diagonal approach to cleft care would foster research experience for local trainees and practitioners in countries where its importance may not be emphasized during medical education. Although a majority of plastic surgeons and volunteer pediatric surgeons express their desire to teach clinical skills to colleagues in LMICs, research skills are largely neglected on mission trips. Relatively few (40%) cleft mission organizations regard research as a priority, citing limited funding, manpower, and time [58]. Research is particularly difficult given the heavy operative census and short duration of medical missions. Transitioning away from a strictly vertical model toward a diagonal model could promote research in several ways. For instance, the diagonal goal of increased surgical capacity would reduce the “backlog” of patient need, and lengthier visits and prolonged collaborations between local and visiting providers could alleviate pressure to operate on as many cleft patients as possible during a short mission. As has been suggested by general surgeons and other surgical subspecialists [79], such changes would allow additional time for research planning and execution.

A greater emphasis on global surgery research is important for several reasons. Surgeons in LMICs self-report a need for increased research training and skills, in addition to clinical assistance [80]. Additionally, improving the quality of particular treatments in LMICs, such as cleft surgeries, requires a better understanding of the local needs, barriers to care, outcomes, and predictive factors that define cleft anomalies and other diseases in those countrie, and which may be unique to resource-limited settings. Finally, promoting research partnerships with local providers and investigators could empower local healthcare professionals to take a more active role in determining how best to address the healthcare needs of their populations.

International research partnerships as a component of diagonal development would also challenge researchers in wealthy countries to address previously neglected research topics. For instance, despite the long-accepted model of coordinated care for CLP [51, 53, 81], there is relatively little comparative outcomes research of cleft care in wealthy countries. In order to identify and learn from the weaknesses of current cleft care models, the global cleft community must incorporate research and research capacity building into care delivery models in all settings. Thus, diagonal development can foster research-driven local practice, outcomes-driven quality improvements, and data-driven infrastructure development, for the benefit of all surgical patients.

### 3.6. Increased Financial Sustainability

Although the cost effectiveness of surgical treatment of cleft palate is well demonstrated by analytic models [15, 16, 82], medical missions may not optimize the return on relatively scarce financial investments in cleft care, in terms of value to individual patients and local communities. The significant financial overhead of medical missions may ultimately detract resources from the patients who need those resources the most. Dupuis [71] estimate that $920 US could be saved, per surgery, if operations were performed by local providers instead of by volunteers from abroad. Additionally, analytic models may not take into account the negative externalities of vertical interventions. While transitioning to a diagonal approach may initially increase some costs, by investing in health systems, infrastructure, human capital, and research capabilities, the diagonal approach can increase the overall value of cleft treatments in LMIC. Indeed, just as domestic programs must justify their value proposition to society [83], the international cleft treatment community must increasingly demonstrate to public and private funders that cleft care investments yield sizable long-term benefits for patients and communities alike. Demonstrating increased capacity could attract funders to global surgery because it offers a superior “return on investment,” both financially and ethically. Looking ahead, diagonal approaches can also move local institutions toward the goal of self-sustained revenue streams by promoting increased patient engagement, health systems development, and government involvement in healthcare.

## 4. Future Directions

### 4.1. Implications for Global General Surgical Capacity

To reiterate, diagonal development turns attention away from importing clinical resources and services, emphasizing deliverables that not only increase capacity for CLP surgery, but also enrich the surgical ecosystem as a whole. Under the current vertical cleft care mission models, surgeons and trainees make significant contributions, but may not increase local capacity to address those surgical diseases that account for the majority of mortality and morbidity: obstetrical complications, trauma, and acute abdominal emergencies [84]. Just as the capacity to provide cleft lip and palate treatment is necessarily affected by the overall shortages in operating theatres and supplies, the converse holds true: an emphasis on diagonal development would yield infrastructure, manpower, and self-sustaining revenue that could have positive implications for treatment of other surgical diseases. In this way, a diagonal approach makes the “spill-over” effect a primary aim, rather than a welcome positive externality.

### 4.2. Advocacy and Implementation

The full extent of surgical care needed in resource-limited settings cannot be addressed by global plastic surgeons alone. However, what plastic surgeons can directly do is very powerful—the transformation of a face or the reconstruction of an injury is a metaphor for involvement creating change, which can reach exponentially to more patients [18]. Each specialty must work both within and outside of the global health community both to offer its expertise—whether that is caesarian sections or CLP repair—and also assist in the development of comprehensive surgical care delivery services, not merely concentrate on a specific disease or intervention [9]. Medical mission NGOs have taken the lead in these efforts; it is now incumbent upon academic surgeons, trainees, and researchers to join medical missions groups in further defining and promoting the global surgery agenda.

## 5. Conclusions

Global surgery is still, in many ways, in its infancy. As we move forward in global CLP care, it is essential to learn from the strengths and limitations of the vertical and horizontal approaches in order to maximize the benefit of these programs to healthcare systems in LMICs. We recognize an ongoing need for vertical humanitarian missions and admire the legacy of many cleft treatment groups. Indeed, it is because of the successes of cleft missions that the cleft care community is now in the position to contribute diagonally to increase surgical capacity and promote quality of care. As funding becomes increasingly available for global surgery interventions, care delivery methods will come to the forefront in terms of achieving optimal outcomes. At this critical juncture, plastic surgeons must serve as thought leaders in global surgery, and a diagonal approach to CLP care is a means of achieving that goal.

## Figures and Tables

**Table 1:** The vertical-horizontal debate: benefits and limitations of each approach.

Approach	Description	Examples	Advantages	Disadvantages
Vertical	(i) Disease specific (ii) Narrowly focused (iii) Operates outside the existing healthcare structures and systems (iv) Often privately funded	(i) Polioimmunization program (ii) HIV/AIDS treatment programs (iii) Male circumcision programs (iv) DOTS	(i) Demonstrated effectiveness (HIV/AIDS) (ii) May have a limited, positive impact on other areas of healthcare delivery (“spill over”) (iii) Fast implementation (iv) Scalable (v) Donor attractiveness (vi) Efficient delivery of disease, specific equipment and supplies	(i) May not address other diseases, healthcare needs, and health determinants (ii) May yield redundant and poorly coordinated efforts (iii) May divert funds from other diseases and medical priorities

Horizontal	(i) Not disease specific (ii) Focuses on broadly applicable healthcare infrastructure (iii) Long-term interventions and investments	(i) Strengthening primary care systems (ii) Healthcare provider education and training (iii) Human resources for health (HRH)	(i) Strengthens health systems as a whole (ii) Benefits all patients, regardless of disease or diagnosis (iii) May facilitate disease, specific treatments (iv) Builds capacity for long-term change	(i) Long-term interventions (ii) Large, unwieldy projects (iii) Often less attractive to donors, funders (iv) Require functional state and local governments (v) More difficult to measure impact of horizontal interventions

**Table 2:** Superior impact of diagonal interventions in global cleft lip and palate care.

	Vertical approach	Diagonal approach

Continuity of care	Short-term interventions	Long-term presence
Interdisciplinary care	Focus on cleft surgery services	Focus on surgical, perioperative, dental, feeding, hearing, speech, and rehabilitation services
Equitable access	Service-driven patient selection	Needs-driven patient selection
Outcomes monitoring	Postoperative	Long term
Local workforce development	Unilateral exchange focusing on cleft surgeons	Bilateral exchange focusing on surgeons, anesthesiologists, surgical intensivists, scrub technicians, perioperative nurses, ward nurses, dentists, feeding specialists, speech therapists, and audiologists
Equitable trainee experiences	Enhanced visiting trainee experience in specialized surgical practice	Enhanced visiting trainee experience in global healthcare delivery; enhanced local trainee experience in surgical practice
Academic culture of investigation and empowerment	Clinical emphasis, transfer of clinical skills, data collection and analysis by visiting providers, research-driven medical missions	Academic emphasis, transfer of research skills, data collection and analysis by local providers, and research-driven local practice
Increased financial sustainability	Dependence on external funding; return on investment may not be optimal	Goal of self-sustained revenue streams; emphasis on increasing ethical, fiscal and systems-wide returns on investments
Implications for local general surgical capacity	“Spill-over” as a welcome positive externality	“Spill-over” as a primary objective
